# Clinical characteristics analysis of immune checkpoint inhibitors-related myocarditis

**DOI:** 10.3389/fimmu.2026.1904778

**Published:** 2026-08-18

**Authors:** Rui Wang, Cuijun Hao, Jiaqi Liu, Zhanshuai Zhang, Shaoqiang Qin

**Affiliations:** 1 Department of Cardiology, the First Affiliated Hospital of Hebei North University, Zhangjiakou, China; 2 Graduate School, Hebei North University, Zhangjiakou, China

**Keywords:** clinical characteristics, immune checkpoint inhibitors, immune-related adverse events, myocarditis, prognosis, severe disease

## Abstract

**Objective:**

To investigate the clinical characteristics of immune checkpoint inhibitors (ICIs)-related myocarditis and the differences between mild and severe cases, aiming to provide evidence for early identification and stratified management.

**Methods:**

A total of 74 patients diagnosed with ICIs-related myocarditis at our hospital from April 2020 to April 2024 were retrospectively enrolled. According to the *Chinese Expert Consensus on the Surveillance and Management of Immune Checkpoint Inhibitor-Related Myocarditis (2020 Edition)* and CTCAE 5.0 criteria, patients were divided into mild group (n=49) and severe group (n=25). The two groups were compared in terms of demographic characteristics, clinical manifestations, laboratory tests, auxiliary examinations, concomitant irAEs, treatment, and outcomes.

**Results:**

The time of onset in the severe group was significantly earlier than that in the mild group [23 days (18, 85) vs. 80 days (48, 132), P = 0.004]. Severe group more frequently presented with multisystem involvement: incidences of myasthenia (56.0% vs. 6.1%), dyspnea (24.0% vs. 0), and ptosis (16.0% vs. 0) were significantly higher (all P<0.05). Levels of cardiac biomarkers (cTnT/I, CK, NT-proBNP) and liver enzymes (ALT, AST) were markedly elevated (all P<0.01). Electrocardiographic ST-T segment changes (56.0% vs. 10.2%) and conduction blocks (32.0% vs. 4.1%) were more common in the severe group. Echocardiography showed higher rates of regional wall motion abnormality (28.0% vs. 6.1%) and pericardial effusion (28.0% vs. 6.1%) in the severe group. The severe group also had higher rates of concomitant myositis (68.0% vs. 40.8%), hepatitis (28.0% vs. 6.1%), and neurotoxicity (32.0% vs. 2.0%) (all P<0.05). Regarding treatment, 100% of the severe group received glucocorticoids (vs. 28.6%), 60.0% required intravenous immunoglobulin, and 28.0% needed respiratory support. Regarding outcomes, all patients in the mild group improved, while the mortality rate in the severe group was 32.0% (8/25).

**Conclusion:**

Patients with severe ICIs-related myocarditis are characterized by early onset, multisystem involvement, marked elevation of cardiac enzymes, prominent electrocardiographic/echocardiographic abnormalities, frequent concomitant irAEs, and high mortality. Clinicians should be highly vigilant for patients presenting with early myasthenia, conduction blocks, or markedly elevated CK/cTn, and promptly initiate intensified immunosuppressive therapy to improve prognosis.

## 1 Introduction

In recent years, immune checkpoint inhibitors (ICIs) have achieved breakthrough progress in the treatment of various malignancies, significantly prolonging patient survival (1). However, with their widespread use, immune-related adverse events (irAEs) have drawn increasing attention. Among these, although ICIs-related myocarditis has a relatively low incidence (approximately 0.1%–1.2%), its insidious onset, rapid progression, and high fatality rate (up to 40%–50%) have made it a severe irAE that requires urgent clinical attention (2, 3).

Current studies indicate that ICIs-related myocarditis can manifest as a continuous spectrum ranging from asymptomatic cardiac enzyme elevation to fulminant heart failure or even sudden death, making early recognition difficult, and specific biomarkers or imaging features are lacking (4, 5). Although the Chinese Expert Consensus on the Surveillance and Management of Immune Checkpoint Inhibitor-Related Myocarditis (2020 Edition) (6) and international guidelines (e.g., the 2022 ESC Cardio-Oncology Guidelines) (7) have proposed diagnostic and grading criteria, systematic studies on the differences in clinical characteristics between mild and severe cases remain scarce. In particular, which clinical manifestations, laboratory indicators, or comorbidities can serve as early warning signals for severe disease in the real-world setting remain unclear (6, 7).

Notably, some studies suggest that ICIs-related myocarditis often coexists with other irAEs (such as myositis and neurotoxicity), forming a “myocarditis-myositis-myasthenia gravis” syndrome, and such patients experience more severe illness and worse prognosis (8–10). Furthermore, whether the time of onset, dynamic changes in cardiac biomarkers, and abnormal electrocardiographic/echocardiographic patterns are associated with disease severity remains to be further validated.

Therefore, based on a single-center retrospective cohort, this study systematically compares the clinical characteristics, laboratory and imaging findings, concomitant irAE profiles, treatment, and outcomes between mild and severe ICIs-related myocarditis, aiming to identify risk factors for severe disease and provide evidence for early warning, risk stratification, and individualized intervention.

## 2 Methods

### 2.1 Study population

This study was a single-center retrospective cohort study. We enrolled patients with malignant tumors who received immune checkpoint inhibitor (ICI) therapy and attended the Department of Oncology, Department of Cardiology, or the Multidisciplinary Cardio-Oncology Clinic of our hospital between April 2020 and April 2024. Patients diagnosed with ICIs-related myocarditis after clinical evaluation and multidisciplinary team (MDT) discussion were included.

In our institution, the initial suspicion of ICIs-related myocarditis was typically raised by the treating oncologists when patients developed new cardiac symptoms (e.g., chest tightness, dyspnea, palpitations, fatigue) or when routine cardiac biomarker monitoring revealed elevated troponin levels during ICI therapy. Upon suspicion, oncologists promptly referred patients to the Department of Cardiology for comprehensive cardiovascular evaluation, including serial troponin measurements, 12-lead ECG, and transthoracic echocardiography. All suspected cases were then discussed in the MDT conference, which included cardiologists, oncologists, radiologists, and rheumatologists, to establish the final diagnosis of ICIs-related myocarditis after excluding other potential etiologies.

Inclusion criteria: (1) Pathologically or cytologically confirmed malignant tumor and receipt of at least one cycle of ICI therapy (including PD-1 inhibitors, PD-L1 inhibitors, or CTLA-4 inhibitors, as monotherapy or combination regimens); (2) Development of cardiac-related clinical manifestations and/or laboratory abnormalities during ICI treatment or after drug discontinuation, with a definite diagnosis of ICIs-related myocarditis established by MDT discussion; (3) Complete clinical data and a follow-up period of at least 3 months from the date of myocarditis diagnosis.

Exclusion criteria: (1) Myocarditis clearly caused by other etiologies, such as viral or bacterial myocarditis, severe infection, or sepsis. Patients with elevated cardiac biomarkers attributable to acute coronary syndrome were also excluded after comprehensive evaluation including coronary angiography or coronary CT angiography when clinically indicated; (2) Persistent elevation of cardiac biomarkers or a definite diagnosis of myocarditis before ICI treatment; (3) Missing key clinical data (e.g., cardiac troponin, electrocardiogram, echocardiography) that prevented severity grading of myocarditis.

### 2.2 Diagnostic criteria and severity grading

The diagnosis of ICIs-related myocarditis followed the diagnostic algorithm recommended by the Chinese Expert Consensus on the Surveillance and Management of Immune Checkpoint Inhibitor-Related Myocarditis (2020 Edition) (6) and the European Society of Cardiology (ESC) 2022 Guidelines on Cardio-Oncology (7). All of the following conditions had to be met: (1) Newly elevated cardiac biomarkers (cardiac troponin T or I above the upper limit of normal); (2) With or without cardiac-related symptoms (e.g., chest tightness, palpitations, dyspnea, fatigue); (3) New electrocardiographic abnormalities (e.g., ST-T changes, conduction blocks, various arrhythmias); (4) Echocardiographic or cardiac magnetic resonance imaging findings suggestive of myocardial structural or functional abnormalities, after excluding other non-immune causes of cardiac injury. The final diagnosis was confirmed by an MDT composed of oncologists, cardiologists, radiologists, and rheumatologists.

According to the above consensus and the Common Terminology Criteria for Adverse Events (CTCAE) Version 5.0, ICIs-related myocarditis was classified into four grades: Subclinical myocardial injury: Elevated troponin only, no symptoms, no electrocardiographic or imaging abnormalities. Mild myocarditis: Elevated troponin with mild symptoms (e.g., slight chest tightness, fatigue); minor electrocardiographic or echocardiographic abnormalities may be present; no need for specific intervention. Severe myocarditis: Markedly elevated troponin with obvious cardiac symptoms (e.g., dyspnea, chest pain, arrhythmias); significant electrocardiographic abnormalities (e.g., high-grade atrioventricular block, sustained ventricular tachycardia); or newly decreased left ventricular ejection fraction (LVEF) <50% on echocardiography; active intervention required. Critical myocarditis: Fulminant myocarditis requiring circulatory support, mechanical ventilation, or extracorporeal membrane oxygenation (ECMO).

In this study, subclinical myocardial injury and mild myocarditis were combined into the mild group, while severe and critical myocarditis were combined into the severe group. Patients in the severe group had to meet at least one of the following criteria: (1) Need for non-invasive/invasive mechanical ventilation or vasoactive drugs to maintain circulation; (2) Malignant arrhythmias (e.g., sustained ventricular tachycardia, ventricular fibrillation) or high-grade/complete atrioventricular block requiring temporary pacemaker intervention; (3) Rapidly progressive heart failure or cardiogenic shock; (4) Death directly attributable to ICIs-related myocarditis. This grouping criteria referenced the definition of grade 3-4 cardiac adverse events in CTCAE 5.0.

### 2.3 Data collection

The following information was systematically collected from the hospital electronic medical record system and outpatient follow-up records:

General information: Age, sex, smoking history (never, former, current), long-term alcohol consumption, comorbidities (hypertension, diabetes mellitus, coronary artery disease, arrhythmias, thyroid disease, liver or kidney dysfunction, etc.).

Tumor and treatment information: Primary tumor type, type of ICIs and treatment regimen (monotherapy or combination with chemotherapy, targeted therapy, radiotherapy, etc.), number of ICI cycles, and time from first ICI administration to myocarditis diagnosis (days).

Definition of time of onset: In this study, the time of onset was defined as the interval (in days) from the first administration of ICIs to the date when the diagnosis of ICIs-related myocarditis was established. The date of diagnosis was determined as the day when cardiac troponin was first documented to be above the upper limit of normal in the context of compatible clinical and/or electrocardiographic findings, following the diagnostic algorithm recommended by the consensus and guidelines (6, 7).

Clinical manifestations: Chest tightness, dyspnea, palpitations, fatigue, myasthenia, chest pain, edema, and the temporal sequence of symptoms.

Laboratory tests: At the time of myocarditis diagnosis and during treatment: cardiac troponin T/I (cTnT/cTnI), N-terminal pro-B-type natriuretic peptide (NT-proBNP), creatine kinase (CK), creatine kinase-MB (CK-MB), myoglobin (MYO), D-dimer, C-reactive protein (CRP), etc. Normal reference ranges and assay methods were recorded.

Auxiliary examinations.

Electrocardiogram (ECG): T-wave changes, ST-T segment changes, conduction blocks, premature contractions, sinus tachycardia/bradycardia, atrial fibrillation, QTc interval prolongation, etc.

Echocardiography: LVEF, structural abnormalities (chamber enlargement, wall thickening), regional wall motion abnormalities, valvular regurgitation, pericardial effusion, elevated pulmonary artery pressure, etc.

Concomitant irAEs: Immune-related myositis, hepatitis, thyroid dysfunction, adrenal insufficiency, pneumonitis, neurotoxicity, etc. Diagnoses were based on corresponding specialty consensus or comprehensive clinical judgment.

Treatment and outcomes: Whether ICIs were discontinued; glucocorticoid use (starting dose, peak dose, cumulative dose, and course); intravenous immunoglobulin; other immunosuppressants (e.g., mycophenolate mofetil, tacrolimus, tofacitinib); supportive care (antiarrhythmic therapy, myocardial nutrition, circulatory support, etc.). Follow-up was at least 3 months. Outcomes were classified as: Improved: Symptoms and laboratory/imaging abnormalities returned to normal or markedly improved, no need for further escalation of therapy. Not improved: Partial improvement of symptoms or indicators, still requiring ongoing treatment. Death.

### 2.4 Ethics statement

The study was approved by the Ethics Committee of the First Affiliated Hospital of Ningbo University (Approval No.: 2026-R272-01, dated 2026-07-23) and followed the Declaration of Helsinki. Informed consent was waived owing to the retrospective design and use of anonymized clinical data.

### 2.5 Statistical analysis

Statistical analysis was performed using SPSS 26.0 software (IBM Corp., Armonk, NY, USA). Normally distributed continuous variables were expressed as mean ± standard deviation ( ± s) and compared between groups using the independent-sample t-test. Non-normally distributed continuous variables were expressed as median (interquartile range) [M (Q1, Q3)] and compared using the Mann-Whitney U test. Categorical variables were expressed as number (percentage) and compared using the χ² test; Fisher’s exact test was used when the expected frequency was less than 5. A two-sided P < 0.05 was considered statistically significant.

## 3 Results

### 3.1 Baseline demographics and clinical characteristics

A total of 74 patients with ICIs-related myocarditis were included in this study, including 49 (66.2%) in the mild group and 25 (33.8%) in the severe group. There were no significant differences between the two groups in age, sex, smoking history, long-term alcohol consumption, or comorbidities such as hypertension, diabetes mellitus, and coronary artery disease (all P>0.05) (**Table 1**). The number of ICI cycles also showed no significant difference between the two groups (P = 0.173). Notably, the median time of onset in the severe group was significantly earlier than that in the mild group [23 days (18, 85) vs. 80 days (48, 132), Z=-2.845, P = 0.004]. Regarding tumor types, the most common primary malignancies in the entire cohort were lung cancer (37.8%, 28/74), followed by esophageal cancer (18.9%, 14/74), gastric cancer (13.5%, 10/74), and melanoma (8.1%, 6/74), with the distribution between the two groups. Regarding ICI regimens, PD-1 inhibitors (including sintilimab, pembrolizumab, nivolumab, and camrelizumab) were used in 85.1% (63/74) of patients, PD-L1 inhibitors in 8.1% (6/74), and combination therapy with anti-PD-1 plus anti-CTLA-4 in 6.8% (5/74). The combination therapy group had a higher proportion of severe myocarditis (3/5, 60.0%) compared with monotherapy groups (22/69, 31.9%), though the difference did not reach statistical significance (P = 0.317) (**Table 1**).

**Table 1:** Comparison of baseline characteristics between the two groups of patients with ICIs-related myocarditis.

Characteristic	Mild group (n=49)	Severe group (n=25)	χ²/Z/t	P
Age (years, xχ ± s)	63.2 ± 11.4	64.5 ± 8.9	0.512	0.61
Sex [n (%)]			0.008	0.928
Male	37 (75.5)	19 (76.0)		
Female	12 (24.5)	6 (24.0)		
Smoking history [n (%)]			4.892	0.087
Never	33 (67.3)	13 (52.0)		
Former	12 (24.5)	8 (32.0)		
Current	4 (8.2)	4 (16.0)		
Long-term alcohol use [n (%)]			2.657	0.103
No	44 (89.8)	19 (76.0)		
Yes	5 (10.2)	6 (24.0)		
Hypertension [n (%)]	20 (40.8)	9 (36.0)	0.162	0.687
Diabetes mellitus [n (%)]	7 (14.3)	2 (8.0)	0.624	0.43
Coronary artery disease [n (%)]	4 (8.2)	5 (20.0)	2.074	0.15
Primary tumor type [n (%)]				
Lung cancer	18 (36.7)	10 (40.0)		
Esophageal cancer	10 (20.4)	4 (16.0)		
Gastric cancer	7 (14.3)	3 (12.0)		
Melanoma	3 (6.1)	3 (12.0)		
Colorectal cancer	3 (6.1)	1 (4.0%)		
Renal cell carcinoma	2 (4.1)	1 (4.0)		
Breast cancer	2 (4.1)	1 (4.0)		
Head and neck cancer	2 (4.1)	1 (4.0)		
Hepatocellular carcinoma	1 (2.0)	1 (4.0)		
Others	1 (2.0)	0		
ICI regimen [n (%)]				
PD-1 inhibitor	43 (87.8)	20 (80.0)		
PD-L1 inhibitor	4 (8.2)	2 (8.0)		
Anti-PD-1 + anti-CTLA-4	2 (4.1)	3 (12.0)		
Number of ICI cycles [M(Q1,Q3)]	3 (2, 5)	2 (1, 4)	-1.362	0.173
Time of onset [d, M(Q1,Q3)]	80 (48, 132)	23 (18, 85)	-2.845	0.004

Data for primary tumor type and ICI regimen are presented for descriptive purposes; statistical comparisons were not performed for these subgroups due to limited sample sizes in certain categories.

### 3.2 Clinical manifestations

Patients in the severe group more often presented with symptomatic myocarditis. In the mild group, 55.1% (27/49) were asymptomatic, whereas only 16.0% (4/25) in the severe group were asymptomatic (P = 0.001). The incidences of myasthenia (56.0% vs. 6.1%), chest tightness (36.0% vs. 12.2%), fatigue (28.0% vs. 8.2%), ptosis (16.0% vs. 0), and dyspnea (24.0% vs. 0) were significantly higher in the severe group (all P<0.05). Moreover, the proportion of patients presenting with ≥2 symptoms was significantly higher in the severe group (60.0% vs. 14.3%, P<0.001) (**Table 2**).

**Table 2:** Comparison of clinical manifestations between the two groups [n (%)].

Manifestation	Mild group (n=49)	Severe group (n=25)	χ²	P
Asymptomatic	27 (55.1)	4 (16.0)	10.482	0.001
Chest tightness	6 (12.2)	9 (36.0)	5.891	0.015
Myasthenia	3 (6.1)	14 (56.0)	21.738	<0.001
Tachypnea	5 (10.2)	4 (16.0)	0.528	0.468
Fatigue	4 (8.2)	7 (28.0)	5.064	0.024
Palpitations	4 (8.2)	2 (8.0)	0.001	0.978
Myalgia	3 (6.1)	5 (20.0)	3.232	0.072
Ptosis	0	4 (16.0)	—	0.010*
Dyspnea (shortness of breath)	0	6 (24.0)	—	0.001*
Chest pain	1 (2.0)	1 (4.0)	0.231	0.631
Edema	1 (2.0)	0	—	1.000*
≥2 symptoms	7 (14.3)	15 (60.0)	16.866	<0.001

*Fisher’s exact test. Tachypnea was defined as a respiratory rate >20 breaths per minute, whereas dyspnea was defined as the patient’s subjective sensation of breathing discomfort.

### 3.3 Laboratory findings

Markers of myocardial injury and systemic inflammation were significantly elevated in the severe group. The median levels of cardiac troponin (cTnT/cTnI), NT-proBNP, CK, CK-MB, ALT, AST, and LDH were all significantly higher than those in the mild group (all P<0.01). The median cTnT/cTnI reached 0.862 μg/L (vs. 0.108 μg/L), and the median CK reached 2745 U/L (vs. 182 U/L) (**Table 3**). No significant difference was observed in D-dimer levels between the two groups (P = 0.350).

**Table 3:** Comparison of laboratory parameters between the two groups [M(Q1,Q3)].

Parameter	Mild group (n=49)	Severe group (n=25)	Z	P
cTnT/cTnI (μg/L)	0.108 (0.047, 0.192)	0.862 (0.335, 4.286)	-5.617	<0.001
NT-proBNP (ng/L)	238 (41, 768)	1108 (378, 1726)	-3.208	0.001
CK (U/L)	182 (92, 215)	2745 (1285, 6198)	-3.972	<0.001
CK-MB (U/L)	25 (21, 34)	85 (46, 265)	-3.884	<0.001
CK-MM (U/L)	110 (74, 172)	1226 (48, 6421)	-1.996	0.046
D-dimer (mg/L)	1.15 (0.62, 3.52)	1.52 (0.85, 3.48)	-0.934	0.35
ALT (U/L)	29 (16, 46)	248 (54, 381)	-3.452	0.001
AST (U/L)	31 (19, 49)	232 (68, 419)	-3.668	<0.001
LDH (U/L)	212 (167, 280)	1358 (1072, 1615)	-4.118	<0.001

### 3.4 Electrocardiographic and echocardiographic Findings

ECG abnormalities were more common and more severe in the severe group. ST-T segment changes (56.0% vs. 10.2%) and conduction blocks (32.0% vs. 4.1%) were significantly more frequent in the severe group (both P<0.01), while a completely normal ECG was found in 32.7% of the mild group but only 8.0% of the severe group (P = 0.019) (**Table 4**).

**Table 4:** Comparison of ECG abnormalities between the two groups [n (%)].

ECG abnormality	Mild group (n=49)	Severe group (n=25)	χ²	P
T-wave changes	11 (22.4)	7 (28.0)	0.281	0.596
ST-T segment changes	5 (10.2)	14 (56.0)	17.942	<0.001
Frequent premature contractions	8 (16.3)	5 (20.0)	0.158	0.691
Sinus tachycardia	7 (14.3)	3 (12.0)	0.075	0.784
Conduction block	2 (4.1)	8 (32.0)	11.278	0.001
Atrial fibrillation	4 (8.2)	2 (8.0)	0.001	0.979
Sinus bradycardia	0	3 (12.0)	—	0.038*
QTc interval prolongation	1 (2.0)	2 (8.0)	1.574	0.21
Ventricular tachycardia	0	2 (8.0)	—	0.102*
Normal ECG	16 (32.7)	2 (8.0)	5.498	0.019

*Fisher’s exact test.

Echocardiography showed that although the median LVEF was within the normal range in both groups, the severe group had a slightly lower LVEF [63% (58, 65) vs. 66% (63, 69), P = 0.012], and significantly higher rates of regional wall motion abnormality (28.0% vs. 6.1%), pericardial effusion (28.0% vs. 6.1%), and elevated pulmonary artery pressure (16.0% vs. 0) (all P<0.05). A completely normal echocardiogram was found in 63.3% of the mild group vs. 32.0% of the severe group (P = 0.010) (**Table 5**). It should be noted that in the severe group, although the median LVEF remained within the normal range (63%), this does not preclude the presence of severe myocarditis. The majority of severe patients (92.0%, 23/25) presented with LVEF ≥50%, yet they met the severity criteria based on other manifestations such as malignant arrhythmias, conduction blocks requiring pacemaker intervention, or the need for vasoactive support.

**Table 5:** Comparison of echocardiographic findings between the two groups.

Echocardiographic parameter	Mild group (n=49)	Severe group (n=25)	χ²/Z	P
LVEF [%, M(Q1,Q3)]	66 (63, 69)	63 (58, 65)	-2.506	0.012
LVEF <50% [n (%)]	0	2 (8.0)	—	0.102*
Structural abnormality [n (%)]	8 (16.3)	5 (20.0)	0.156	0.693
Regional wall motion abnormality [n (%)]	3 (6.1)	7 (28.0)	6.826	0.009
Valvular regurgitation [n (%)]	9 (18.4)	6 (24.0)	0.328	0.567
Elevated pulmonary artery pressure [n (%)]	0	4 (16.0)	—	0.010*
Pericardial effusion [n (%)]	3 (6.1)	7 (28.0)	6.667	0.01
Normal echocardiogram [n (%)]	31 (63.3)	8 (32.0)	6.550	0.01

LVEF, left ventricular ejection fraction; *Fisher’s exact test.

### 3.5 Concomitant other immune-related adverse events

The severe group was more likely to have other concomitant irAEs. Myositis (68.0% vs. 40.8%, P = 0.028), hepatitis (28.0% vs. 6.1%, P = 0.008), and neurotoxicity (32.0% vs. 2.0%, P<0.001) were significantly more common in the severe group (**Table 6**). No significant differences were found in the incidences of thyroid dysfunction, adrenal insufficiency, or pneumonitis between the two groups (all P>0.05).

**Table 6:** Comparison of concomitant other irAEs between the two groups [n (%)].

Concomitant irAE	Mild group (n=49)	Severe group (n=25)	χ²	P
Myositis	20 (40.8)	17 (68.0)	4.832	0.028
Thyroid dysfunction	21 (42.9)	8 (32.0)	0.832	0.362
Hepatitis	3 (6.1)	7 (28.0)	6.972	0.008
Adrenal insufficiency	5 (10.2)	3 (12.0)	0.054	0.816
Pneumonitis	4 (8.2)	3 (12.0)	0.276	0.599
Neurotoxicity	1 (2.0)	8 (32.0)	14.162	<0.001
At least one other irAE	39 (79.6)	23 (92.0)	1.906	0.167

### 3.6 Treatment and outcomes

All patients in the severe group received glucocorticoid therapy, whereas only 28.6% in the mild group did (P<0.001). The median cumulative glucocorticoid dose in the severe group was 4150 mg, significantly higher than that in the mild group (P<0.001). Moreover, the severe group more frequently required intravenous immunoglobulin (60.0% vs. 6.1%) and other immunosuppressants (32.0% vs. 2.0%) (both P<0.001). Seven patients (28.0%) in the severe group required respiratory support, and two (8.0%) required a temporary pacemaker (**Table 7**).

**Table 7:** Comparison of treatment and outcomes between the two groups [n (%)].

Treatment/Outcome	Mild group (n=49)	Severe group (n=25)	χ²/Z	P
Treatment				
ICIs discontinued	40 (81.6)	25 (100.0)	5.19	0.023
Glucocorticoid therapy	14 (28.6)	25 (100.0)	35.42	<0.001
Cumulative glucocorticoid dose [mg, M(Q1,Q3)]	0 (0, 600)	4150 (1480, 6082)	-5.678	<0.001
Intravenous immunoglobulin	3 (6.1)	15 (60.0)	25.14	<0.001
Other immunosuppressants	1 (2.0)	8 (32.0)	14.078	<0.001
Non-invasive/invasive ventilation	0	7 (28.0)	—	<0.001*
Temporary pacemaker	0	2 (8.0)	—	0.102*
Outcome				
Improved	49 (100.0)	17 (68.0)	15.068	<0.001
Death	0	8 (32.0)	—	<0.001*
Resumption of ICIs	3 (6.1)	0	—	0.548*

*Fisher’s exact test; Improvement was defined as return of symptoms and indicators to normal or marked improvement; other immunosuppressants included tofacitinib, mycophenolate mofetil, tacrolimus, and anti-thymocyte globulin.

Regarding outcomes, all patients in the mild group improved (100.0%), with no deaths. In the severe group, the improvement rate was 68.0% (17/25) and the mortality rate was 32.0% (8/25) (P<0.001). Three patients in the mild group resumed ICIs therapy after recovery from myocarditis, while no patient in the severe group did (**Table 7**).

## 4 Discussion

In this retrospective analysis of 74 patients with ICIs-related myocarditis, we systematically compared the differences between the mild and severe groups in clinical manifestations, laboratory parameters, imaging features, concomitant irAEs, and outcomes. The results showed that severe cases were characterized by earlier onset, prominent multisystem involvement, markedly elevated cardiac enzymes, severe electrocardiographic/echocardiographic abnormalities, a high proportion of concomitant myositis or neurotoxicity, and high mortality, providing important clues for early identification of high-risk patients.

First, the median time of onset in the severe group was 23 days (IQR: 18–85 days), significantly earlier than that in the mild group (80 days, IQR: 48–132 days, P = 0.004), suggesting that the early period of ICIs therapy (especially the first 1–2 months) is a critical window for the development of myocarditis and its progression to severe disease. This finding is consistent with the large-sample study by Mahmood et al. (11), which reported that about 65% of ICIs-related myocarditis occurred within 30 days of the first dose. The study by Salem et al. (12) also showed that more than 80% of myocarditis occurred after the first 1–2 cycles of ICIs. Therefore, clinicians should strengthen the monitoring of cardiac enzymes and electrocardiograms in the early phase of ICIs treatment, and maintain a high degree of vigilance especially for patients presenting with non-specific symptoms such as fatigue, chest tightness, or myalgia.

Second, neuromuscular symptoms including myasthenia (56.0% vs. 6.1%, P<0.001), ptosis (16.0% vs. 0), and dyspnea (24.0% vs. 0) were significantly more frequent in the severe group, strongly suggesting the presence of “myocarditis-myositis-myasthenia gravis overlap syndrome” (MMM overlap syndrome). This overlap syndrome has been shown in several studies to be associated with anti-acetylcholine receptor antibodies or anti-Titin antibodies, and often presents with rapidly progressive arrhythmias, conduction blocks, and even sudden death (13–16). In the present study, the incidence of conduction block in the severe group was as high as 32.0%, significantly higher than that in the mild group (4.1%, P = 0.001), and all eight patients who died had myasthenia or neurotoxicity, further supporting the high lethality of this phenotype. Literature reports indicate that the in-hospital mortality of MMM overlap syndrome can reach 38%–60% (17). Therefore, once a patient develops myasthenia or ptosis during ICIs therapy, even in the absence of typical chest pain, electrocardiography and troponin testing should be performed immediately, and neurophysiological examination and antibody testing should be carried out when necessary.

Third, markedly elevated creatine kinase (CK) and cardiac troponin (cTnT/cTnI) are important laboratory markers of severe myocarditis. In this study, the median CK in the severe group was 2745 (1285, 6198) U/L, and the median cTnT/cTnI was 0.862 (0.335, 4.286) μg/L, both much higher than those in the mild group (both P<0.001). Of note, elevated CK not only reflects myocardial injury but may also originate from skeletal muscle destruction due to concomitant myositis. When CK and cTn are synchronously and markedly elevated, combined myocardial-skeletal muscle injury should be highly suspected, and electromyography or muscle biopsy should be considered for definitive diagnosis (18). In addition, the levels of ALT, AST, and LDH in the severe group were also significantly higher than those in the mild group, indicating that multiple organ involvement may be associated with systemic immune activation. There was no significant difference in D-dimer levels between the two groups (P = 0.350), suggesting that coagulation activation is not a major pathological mechanism of ICIs-related myocarditis, or that its value in severity grading is limited.

With regard to auxiliary examinations, ST-T segment changes and conduction block are electrocardiographic danger signals of severe myocarditis. In this study, ST-T segment changes occurred in 56.0% of severe patients and conduction block in 32.0%, compared with 10.2% and 4.1% in the mild group, respectively. It should be clarified that among the 32.0% of severe patients with conduction block, sinus bradycardia was observed in 12.0% (3/25) and ventricular tachycardia in 8.0% (2/25). However, sinus bradycardia and ventricular tachycardia are not themselves classified as conduction blocks; rather, they represent distinct rhythm disturbances that may coexist with or occur in the context of conduction system abnormalities. The conduction blocks observed in this study primarily included first-degree atrioventricular block, second-degree type I and type II atrioventricular block, and complete atrioventricular block. Conduction block (especially high-grade atrioventricular block) is often the initial manifestation of fulminant myocarditis, progresses rapidly, and requires urgent pacemaker intervention (19). The two patients who required temporary pacemakers in this study were both in the severe group, confirming the importance of dynamic electrocardiographic monitoring. On echocardiography, although the median LVEF was within the normal range in both groups, the severe group had a significantly lower LVEF (63% vs. 66%, P = 0.012), and significantly higher rates of regional wall motion abnormality (28.0% vs. 6.1%, P = 0.009), pericardial effusion (28.0% vs. 6.1%, P = 0.010), and elevated pulmonary artery pressure (16.0% vs. 0, P = 0.010). Notably, the median LVEF of 63% in the severe group, while within the normal range, does not contradict the severity of the condition. As documented in **Table 5**, only two patients (8.0%) in the severe group had LVEF <50%, whereas the majority of severe patients met the severity criteria based on arrhythmic or hemodynamic manifestations rather than systolic dysfunction. This pattern—severe clinical presentation with preserved LVEF—has been previously described in ICIs-related myocarditis and underscores the importance of comprehensive evaluation beyond ejection fraction alone (11, 19). This suggests that a normal LVEF cannot be used to rule out severe myocarditis, and a combination of multiple imaging modalities should be used for dynamic assessment.

Regarding concomitant other irAEs, 83.8% of patients in this study had at least one other irAE, with myositis (50.0%) and thyroid dysfunction (39.2%) being the most common. The proportions of myositis (68.0% vs. 40.8%, P = 0.028), hepatitis (28.0% vs. 6.1%, P = 0.008), and neurotoxicity (32.0% vs. 2.0%, P<0.001) were significantly higher in the severe group. This finding is consistent with domestic and international reports: ICIs-related myocarditis is often accompanied by irAEs in other systems, and multiple organ involvement often predicts a worse prognosis (20–22). Therefore, when an irAE occurs in one organ, clinicians should consciously screen for involvement of other organs, especially the heart, to avoid missed diagnosis.

With respect to treatment and outcomes, all patients in the severe group received glucocorticoid therapy, and the median cumulative glucocorticoid dose was as high as 4150 mg, significantly higher than that in the mild group (P<0.001). The severe group also had significantly higher rates of combination therapy with intravenous immunoglobulin (60.0% vs. 6.1%) and other immunosuppressants (32.0% vs. 2.0%) (both P<0.001), yet the mortality rate remained as high as 32.0% (8/25), highlighting the difficulty of treatment. This mortality rate is consistent with the 30%–46% reported in the literature (23, 24). In contrast, all patients in the mild group improved after glucocorticoid treatment, with no deaths, and three patients with mild myocarditis successfully resumed ICIs therapy after recovery without recurrence of myocarditis. This suggests that for mild patients without high-risk features, ICIs rechallenge may be considered under close monitoring, whereas severe patients require permanent discontinuation and long-term follow-up.

**Figure 1:**
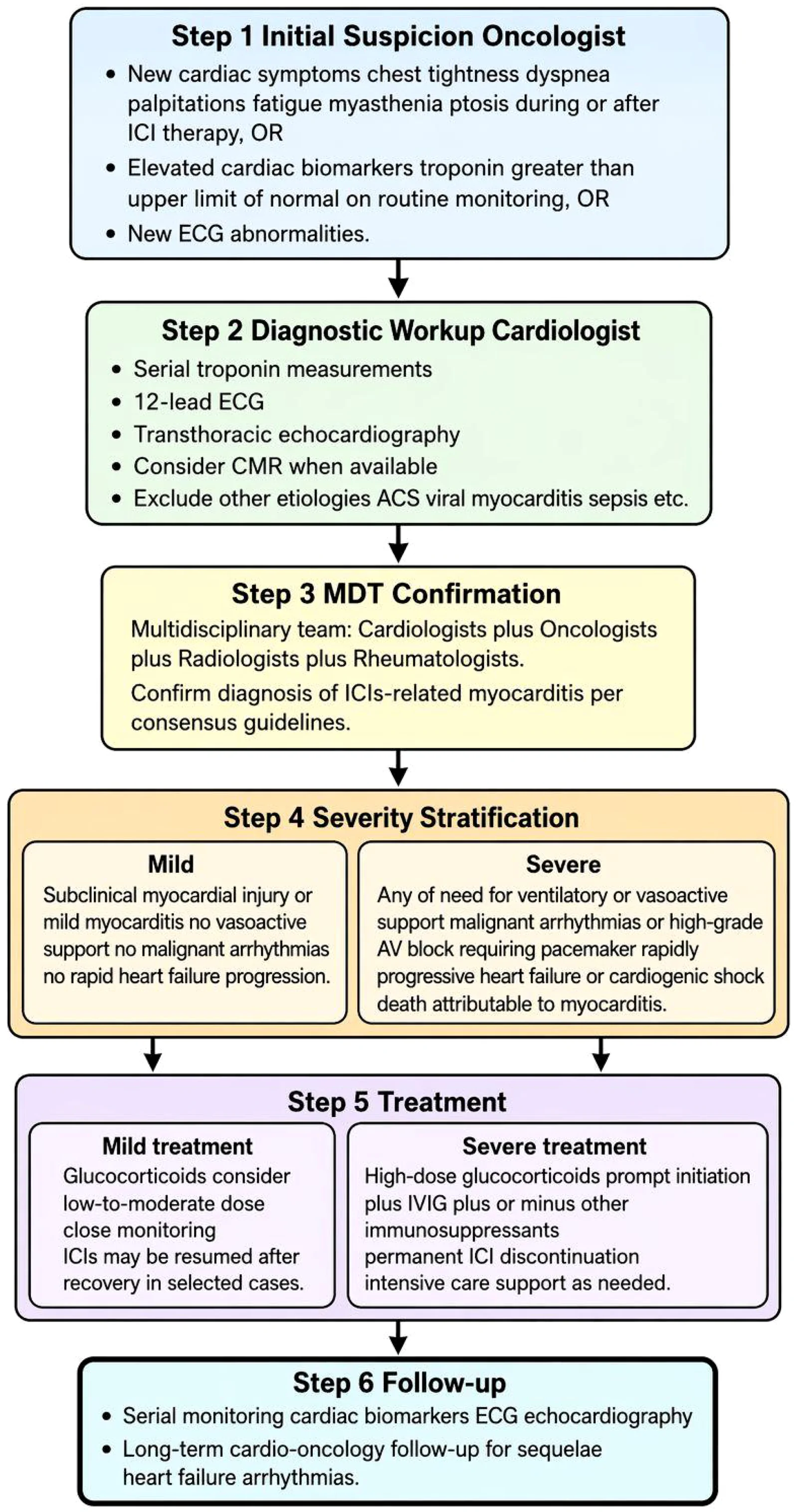
Proposed management algorithm for ICIs-related myocarditis.

Based on the findings of this study and the diagnostic workflow recommended by the Chinese Expert Consensus (6) and ESC guidelines (7), we propose a practical management algorithm for ICIs-related myocarditis (**Figure 1**). The algorithm outlines the steps from initial suspicion (triggered by elevated cardiac biomarkers, new cardiac symptoms, or ECG abnormalities during ICI therapy) through diagnostic confirmation (via MDT discussion incorporating laboratory, ECG, echocardiographic, and when available, CMR findings), severity stratification (mild vs. severe according to the criteria defined in Methods), and therapeutic decision-making (glucocorticoids as first-line therapy, with escalation to IVIG or other immunosuppressants for severe cases, and consideration of rechallenge for mild cases after complete recovery). This algorithm is intended to provide clinicians with a clear, step-by-step framework for the management of this potentially fatal complication (25).

Nevertheless, several limitations should be acknowledged when interpreting our findings. First, it is a single-center retrospective study, and selection bias may exist; the generalizability of the conclusions needs to be validated by multicenter, large-sample studies. Second, cardiac magnetic resonance (CMR) or endomyocardial biopsy was not routinely performed, and some diagnoses relied on comprehensive clinical judgment and multidisciplinary consultation, which may introduce diagnostic bias. Third, the sample size was limited, especially the severe group with only 25 patients and only four cases of MMM overlap syndrome, which may affect the statistical power of some subgroup analyses. Fourth, this study is a pooled analysis based on published literature data; some variables (e.g., cumulative glucocorticoid dose, combination drug regimens) may have differed among centers and were not fully standardized, which may have influenced the results. Fifth, the follow-up period was only three months, and long-term outcomes of myocarditis (such as chronic heart failure and sequelae of arrhythmias) were not fully evaluated. Future prospective, multicenter studies are needed to further explore early predictive biomarkers of ICIs-related myocarditis (such as anti-cardiac antibodies and cytokine profiles) and to optimize treatment strategies.

In conclusion, severe ICIs-related myocarditis is characterized by early onset, prominent neuromuscular symptoms, markedly elevated CK/cTn, electrocardiographic conduction abnormalities, frequent association with myositis and neurotoxicity, high treatment intensity, and high mortality. Clinicians should be highly vigilant for patients presenting with myasthenia, ptosis, conduction block, or synchronously elevated CK/cTn in the early period of ICIs treatment, and promptly initiate multidisciplinary team (MDT) consultation and intensified immunosuppressive therapy to improve prognosis. For mild myocarditis, resumption of ICIs after adequate treatment may be feasible but should be performed under close monitoring.

## Data Availability

The original contributions presented in the study are included in the article/supplementary material. Further inquiries can be directed to the corresponding author.
